# A Cross-sectional Comparative Analysis of Eleven Population Pharmacokinetic Models for Docetaxel in Chinese Breast Cancer Patients

**DOI:** 10.2174/0113892002322494240816032948

**Published:** 2024-08-16

**Authors:** Genzhu Wang, Qiang Sun, Xiaojing Li, Shenghui Mei, Shihui Li, Zhongdong Li

**Affiliations:** 1 Electric Power Teaching Hospital, Capital Medical University, Beijing, 100073, China;; 2 Beijing Tiantan Hospital,*Capital Medical University, Beijing, 100070, China*

**Keywords:** External evaluation, docetaxel, population pharmacokinetics, breast cancer, predictive performance, hematological toxicity

## Abstract

**Objective:**

Various population pharmacokinetic (PPK) models have been established to help determine the appropriate dosage of docetaxel, however, no clear consensus on optimal dosing has been achieved. The purpose of this study is to perform an external evaluation of published models in order to test their predictive performance, and to find an appropriate PPK model for Chinese breast cancer patients.

**Methods:**

A systematic literature search of docetaxel PPK models was performed using PubMed, Web of Science, China National Knowledge Infrastructure, and WanFang databases. The predictive performance of eleven identified models was evaluated using prediction-based and simulation-based diagnostics on an independent dataset (112 docetaxel concentrations from 56 breast cancer patients). The -2×log (likelihood) and Akaike information criterion were also calculated to evaluate model fit.

**Results:**

The median prediction error of eight of the eleven models was less than 10%. The model fitting results showed that the three-compartment model of Bruno *et al.* had the best prediction performance and that the three compartment model of Wang *et al.* had the best simulation effect. Furthermore, although the covariates that significantly affect PK parameters were different between them, seven models demonstrated that docetaxel PK parameters were influenced by liver function.

**Conclusions:**

Three compartment PPK models may be predictive of optimal docetaxel dosage for Chinese breast cancer patients. However, for patients with impaired liver function, the choice of which model to use to predict the blood concentration of docetaxel still requires great care.

## 1 INTRODUCTION

Female breast cancer was the most commonly diagnosed cancer in 2020, with an estimated 2.3 million new cases and 685,000 deaths that year, accounting for 11.7% of all new cancer cases and 6.9% of all new cancer deaths [1, 2]. In China, the incidence and death rates of breast cancer are higher than the rest of the world, which are 43.3 and 9.8 per 100,000 people, respectively [1, 3]. Docetaxel is a first-line drug for the treatment of breast cancer that can promote microtubule polymerization and inhibit its depolymerization, resulting in tumor cell death [4-6]. Since docetaxel is a narrow- treatment-window anti-tumor drug, overdose of the drug may lead to severe hematological toxicity, and underdose may result in treatment failure [4, 7-12]. The most common clinical dosage of docetaxel is 75 mg/m^2^, every 3 weeks [13]. How ever, the pharmacokinetic (PK) parameters of docetaxel show great inter-individual variability, up to 7-fold variation [14, 15]. Hence, the currently used dosing strategy can still be optimized on an individual basis.

Population PK (PPK) is an most important method for personalized treatment [16-19], and numerous studies have already established PPK models of docetaxel [4, 16, 20-25]. However, these models naturally have different predictive capability [8], and none of them have been fully evaluated and verified by an external data set. In our present study, we retrospective analyzed all published docetaxel PPK models and used an external evaluation data set of Chinese breast cancer patients to assess their predictive ability in order to determine a best model for precision dosing of docetaxel.

## 2 MATERIALS AND METHODS

### 2.1 Database and Search Strategy

This systematic review was conducted according to the “Preferred Reporting Items for Systematic Reviews and Meta-Analysis” [26-28]. A systematic search was conducted to identify the literature on population pharmacokinetic analysis of docetaxel using PubMed, Web of Science, WanFang data, and China National Knowledge Infrastructure databases up to July, 2024. The following keywords were used: “docetaxel” (OR) “taxotere” (AND) “population pharmacokinetic” [OR] “pharmacokinetics” (OR) “nonlinear mixed effect model” (AND) “cancer”. Moreover, the references in the selected manuscripts were also manually screened [27].

### 2.2 Inclusion Criteria

Eligible studies were required to satisfy the following criteria: (1) adult cancer patients, (2) intravenous docetaxel administration, and (3) adequate pharmacokinetic parameters for recording the control file. If given models included covariates that were not included in our dataset, we used its basic PPK model for evaluation [21].

### 2.3 Exclusion Criteria

We excluded models if any of the following were true. (1) The model was not suitable for external evaluation for any reason [29], (2) the model was developed based on healthy volunteers, (3) the model was a duplicate search result, or (4) the model was developed based on oral docetaxel administration [30-32].

### 2.4 Data extraction

#### 2.4.1 Pharmacokinetic Studies

The study information and modeling information of the populations were extracted from each of the retrieved studies.

#### 2.4.2 Patients’ Medical Records

The minimum sample size was calculated for external validation as suggested in the previous studies [33-35]. Briefly, the following equation: 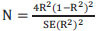 was used to calculate sample size, where R^2^ and SE (R^2^) represent the proportion of variance and the target standard error of the estimated R^2^ in the external validation dataset, respectively. Blood docetaxel concentration data and demographic data were extracted from Electric Power Teaching Hospital. The data included patients’ age, height, body weight, body surface area (BSA), total protein (TP), albumin (ALB), alanine aminotransferase (ALT), aspartate aminotransferase (AST), total bilirubin (TBIL), direct bilirubin (DBIL), and serum creatinine (SCr). This study was approved by the Ethical Committee of Electric Power Teaching Hospital, Capital Medical University (KY-2021-018-01).

### 2.5 Treatment and Data Collection

Docetaxel was administered by intravenous infusion at a dose of 75 mg/m^2^ once every 3 weeks in every case, and two peripheral blood samples (2 mL in an EDTA tube) were collected 5-10 min before the end of infusion and 60 min after the end of infusion. Flexibility in this regimen was allowed depending on the clinical situation. Docetaxel concentrations were determined by HPLC-MS/MS assay [36-41] using the Anticancer Drugs TDM LC-MS/MS Assay Kit (Zhejiang DiSigns Diagnostics, Ltd., Hangzhou, China).

### 2.6 Data Analysis

The Phoenix 8.1.0 (Certara Company, USA) software was used for our external evaluation that included both predictive and simulation-based diagnostics [18, 19, 34, 35, 42]. Dependent variable-individual predicted values (DV-PRED) were obtained to assess the predictive performance of the models, and the prediction error (PE) was calculated to assess the predictive performance of the models using the following equation: PE%=(PRED-DV)/DV*100% [11,19]. Box plots of PE were generated by Graphpad Prism 8.0, and the median prediction error (MPE) was used to evaluate accuracy of the predictive performance [19]. F_20_ and F_50_ indicate that the percentage of PEs fell within ±20% and ±50%, respectively [19]. They were calculated as joint predictors of accuracy and precision [19].

Simulation-based diagnostics were conducted using visual predictive checks (VPCs) that were used to evaluate whether prediction-corrected simulations generated by a candidate model deviated from prediction-corrected observed data [18]. The dataset was simulated 1,000 times, and the VPCs were generated using Phoenix NLME software [18]. The 95% confidence intervals (CIs) for the median and the 5^th^, 50^th^, and 95^th^ percentiles of the simulations at different times were calculated and compared to the observations [19].

## 3 RESULTS

### 3.1 Data

**Fig. (1):**
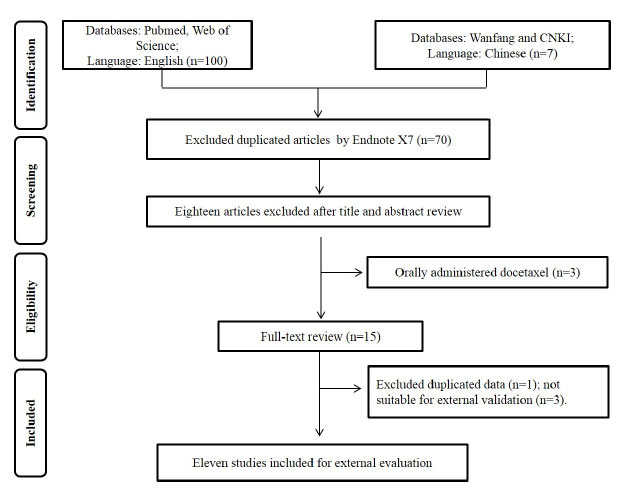
Flowchart of the literature selection process.

Full-text screening process was conducted according to the inclusion and exclusion criteria. Our searches yielded 107 potentially relevant records, and after titles, abstracts, and full texts were thoroughly screened, eleven PPK models of docetaxel were finally included in our study (Fig. **1**). Among these models, four were from China, two were from Japan, two were from France, one was from India, one was from Australia, and one was from the Netherlands. The population and modeling information are summarized in Tables **1** and **2**. Three studies implemented a two-compartment model, and eight studies employed a three-compartment model. Three studies employed an intensive sampling (six or more samples per patient) strategy. Age, body mass index (BMI), BSA, ALB, α1-acid glycoprotein (AAG), SCr, and hepatic function indices (HEP) were the most common covariates. Typical clearance values ranged from 18 to 57.2 L/h, and the typical volume for central compartment ranged from 5.73 to 12.00 L among these studies.

The external evaluation data set included 56 patients with 112 docetaxel concentrations, where the concentrations range from 221-3,430 μg/L and 14.7-375 μg/L at 5-10 min before the end of infusion and 60 min after the end of infusion, respectively. The population baseline characteristics are summarized in Table **3**.

### 3.2 External Predictability Evaluation

The predictive performance of the different models is shown in Fig. (**2**) and Table **4**. All models had an MPE< 0, indicating under-prediction on average (median) of the observed plasma concentrations in the external evaluation data set [35]. Eight models were found to have MPE≤10%, showing good prediction accuracy. However, no established models with MPE close to 0 and F_20_ ≥ 65%, were found, meaning no model had both good predictive accuracy and precision [18]. Bruno *et al.* model with an MPE closest to 0, indicating that this model may have the best accuracy and precision for population estimates [16]. The largest values of F_20_% and F_50_% were obtained in Crombag *et al.* model [24], indicating that this model had the best relative accuracy and precision.

The data from the simulation-based diagnostics are presented in Fig. (**3**) and Table **4**. Although simulated data for the models of Wei *et al.* and Wang *et al.* were within the 95% prediction interval of observed data at the 5^th^ and 50^th^ percentiles, these models under-predicted the external evaluation data at the 95^th^ percentile, indicating slight misspecification of parameter estimates [4, 43]. We additionally calculated the -2×log (likelihood) (-2LL) and Akaike information criterion (AIC) to evaluate model fit. The model of Wang *et al.* had the smallest -2LL and AIC values, giving it the best performance in simulation of docetaxel concentrations [43].

The red solid dashed line represents the observed 50^th^ percentile, and the semitransparent pink field represents the simulation-based CIs for the median. The observed 5^th^ and 95^th^ percentiles are shown with red solid lines and red dotted lines, respectively, and the simulation-based CIs for the corresponding model-predicted percentiles are presented with semitransparent blue fields. The observations are represented by blue dots.

## 4 DISCUSSION

Performing external verification for published PPK models can provide guidance in formulating appropriate dosing regimens that in turn can reduce both adverse reactions, and medical expenses [4, 46-48]. PPK models of docetaxel have been established by lots of studies [49-53]. However, most of them were built with a small population in a single center [21-23, 25, 44]. Their predictive abilities may therefore not be consistent when applied to other centers. In addition, a comprehensive validation and evaluation procedure necessarily includes an internal model evaluation followed by an external evaluation and a prospective clinical study [42]. However, to the best of our knowledge, no docetaxel PPK models have included this complete process.

External evaluation is usually used to assess the reliability of PPK models prediction performance [19]. In this study, we compared the predictive ability of different models to find a suitable docetaxel PPK model for Chinese breast cancer patients specifically. Potential factors contributing to the PK parameters included age, plasma protein concentrations, and hepatic and renal functions [22, 54]. In prediction-based diagnostics, only three models did not have a good degree of fit, with MPE>10% [21, 24, 25], and these three models were all based on non-Chinese populations, suggesting that the underlying pharmacokinetic processes of docetaxel may be associated with racial factors [8, 55-58]. Although some researchers have reported that ethnic differences did not significantly influence docetaxel’s PK profiles [59], we recommended choosing a model that has been validated on the same race as a given patient.

In addition, the weekly docetaxel administration and advanced age of certain patients may also have contributed to the MPE [21, 24]. Among all the models, the one developed by Bruno *et al.* had the minimum MPE (-2.69%) [16], and it also had the maximum sample size and total observations. Furthermore, its average age and BSA of patients was close to the external evaluation population in our present study. Thus, the predictive ability of the model of Bruno *et al.* was the highest for our validation dataset. The model of Slaviero *et al.* had the biggest MPE (-12.26%) [21], but this model contained the covariate (1/_Tmax_), which was not included in our dataset, and a large proportion of elderly patients were also included in their model, so it was not as applicable.

From our simulation-based diagnostics, all models showed poor predictive ability according to the VPC results. Although the models of Wang *et al.* and Wei *et al.* performed relatively well, likely because they featured the same race, a similar dosing regimen, and average SCr and TBIL values close to our external evaluation population. However, we must note their poor 95^th^ observed qualities [4, 43] due to the fact that high blood concentration of docetaxel contributes directly to its hematological toxicity [7]. According to all the diagnostic results, no model performs well in terms of prediction and simulation-based diagnostics. Hence, the transferability of any of these published PPK models must be evaluated before using them in a different clinical setting.

Our study has several advantages relative to the existing literature, the external data set was prospectively collected, which may reduce the risk of data loss and the exclusive use of accurate blood sampling times made our results more reliable. However, this study also has some limitations. First, only two concentrations were tested. Second, some breast cancer patients also received with other agents, such as ritonavir, concurrently with docetaxel, which may have affected docetaxel pharmacokinetics and led to prediction bias [32, 60-62].

## CONCLUSION

Eleven published docetaxel PPK models were evaluated using an independent dataset and these published models indicated that liver function was an important covariate for docetaxel PPK parameters. Furthermore, three-compartment models may be predictive of the ideal blood concentration of docetaxel. However, for patients with impaired liver function, choosing which model to use to make such a prediction in clinical practice should be done with great care.

## Figures and Tables

**Fig. (2):**
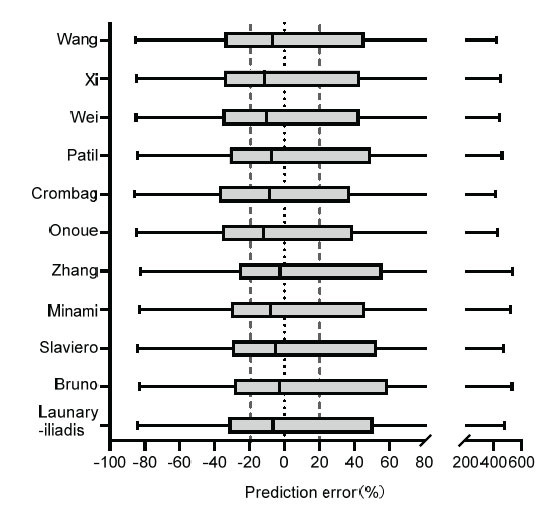
Box plots of prediction error for the candidate models.

**Fig. (3):**
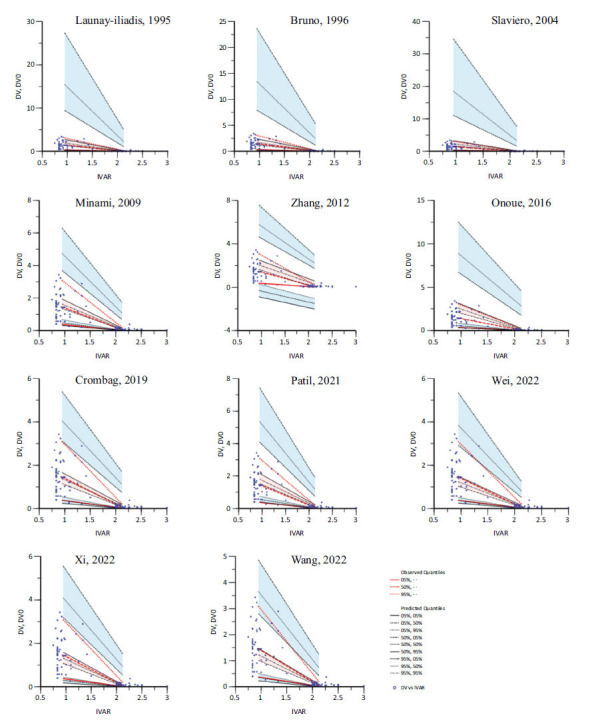
Visual predictive check plots of the candidate models.

**Table 1:** The study information of the populations included in the pharmacokinetic studies.

**Author, Year**	**Region**	**No. of** **Patients**	**No. of** **Observation**	**Age/Year**	**BSA/m^2^**	**Treatment**	**Assays**
Launay-Iliadis *et al.*, 1995 [20]	France	26	389	52.30 (35~65)	1.68 (1.30~2.02)	70~115 mg/m^2^ 3weeks^-1^	HPLC
Bruno *et al.*, 1996 [16]	France	547	1890	56 (39~71)	1.78 (1.47~2.16)	70~115 mg/m^2^ 3weeks^-1^	HPLC
Slaviero., 2004 [21]	Australia	54	145	63 (43~83)	1.49 (1.14~2.27)	55~80 mg/m^2^ weeks^-1^	HPLC
Minami *et al.*, 2009 [22]	Japan	200	1200*	57 (21~86)	1.53 (1.17~1.99)	15~60 mg/m^2^ 3weeks^-1^	HPLC
Zhang *et al.*, 2012 [44]	China	80	210	53.57 (35~76)	1.58 (1.37~1.78)	120 mg/m^2^ 3weeks^-1^	HPLC
Onoue *et al.*, 2016 [23]	Japan	24	142	58 (40~75)	1.49 (1.33~1.67)	40 mg/m^2^ 3weeks^-1^	LCMS/MS
Crombag *et al.*, 2019 [24]	Netherlands	157	380	67 (31~87)	1.9 (1.4~2.3)	15~102 mg/m^2^ 3weeks^-1^	LCMS/MS
Patil *et al.*, 2021[25]	India	48	323	44 (24~79)	1.64 (1.1~2.0)	65~150 mg/m^2^ 3weeks^-1^	HPLC
Wei *et al.*, 2022 [4]	China	440	880	50 (13~79)	1.61 (1.29~2.15)	60~200 mg/m^2^ 3weeks^-1^	ACBA
Xi *et al.*, 2022 [45]	China	374	772	51 (47~57)	NG	75 mg/m^2^ 3weeks^-1^	LCMS/MS and ACBA
Wang *et al.*, 2022 [43]	China	132	264	55.5 (34~88)	1.68 (1.38~2.41)	60~75 mg/m^2^ 3weeks^-1^	ACBA

**Note:** *Number of observations was calculated by the median number of each patient multiplied by 200; **Abbreviations:** LCMS/MS: Liquid chromatography-tandem mass spectrometry; HPLC: high-performance liquid chromatography; ACBA: auto-chemistry biochemical analyzer; NG: not given.

**Table 2:** Modeling information in the included studies.

**Author**	**Compartment** **Model**	**Significant** **Covariates**	**Estimates of PK** ** Parameters**	**Final Model Equations**	**Model** **Evaluation**	**PPK** **Software**	**Residual Valuation /%**
Onoue *et al.* [23]	Two	AGE	CL_1_=32.6, CL_2_ =5.34, V_1_=5.77, V_2_ =11.0	CL=32.6×1.24(>58 years)	VPC and goodness of fit	NONMEM	Proportional/16.4
Slaviero *et al.* [21]	Two	1/_Tmax_, ALT	CL=30.13, V=7.90, K_12_=1.13, K_21_=0.15	CL= 21.51+217/_Tmax_ - 0.13×ALT	Internal validation and VPC	P-PHARM	Proportional/30.0
Wei *et al.* [4]	Two	BMI, BSA, AGE	CL_1_=38.2, CL_2_ =40.99, V_1_=5.73, V_2_ =270.87	CL=38.2×(BMI/24.84)^1.31^× (BSA/1.61)^0.99^×(AGE/50)^-0.72^	VPC and goodness of fit	NONMEM	Proportional/27.3
Zhang *et al.* [44]	Three	BSA, ALB, HEP	CL_1_=25.2, CL_2_=5.17, CL_3_=15.2, V_1_=7.38, V_2_=8.27, V_3_ = 673	CL=25.2×(BSA/1.58)^1.15^× (ALB/3.6)^1.47^×HEP	Bootstrap and goodness of fit	NONMEM	Additive/ 25.6
Minami *et al.* [22]	Three	BSA, ALB, HEP, AAG	CL_1_=29.3, CL_2_=5.46, CL_3_=19.0, V_1_=7.75, V_2_=8.69, V_3_ = 660	CL=29.3×(BSA/1.53)^1.11^× (ALB/3.7)^2.0^×(97/AAG) ^0.251^×LIV×EXP(η1)	Bootstrap and goodness of fit	NONMEM	Proportional/19.0
Bruno *et al.* [16]	Three	BSA, ALB, AGE, HEP, AAG	CL=36.7, V=8.31, Ka=4.42, K_12_=1.07, K_21_=1.74, K_13_=1.28, K_31_=0.0787	CL=BSA× (22.1-3.55×AAG-0.095×AGE+0.225×ALB) (1-0.334×HEP12)	Internal validation and VPC	NONMEM	Proportional/20.5
Launay-iliadis *et al.* [20]	Three	BSA, AGE	CL=35.6, V=5.74, K_12_=1.35, K_21_=1.31, K_13_=1.37, K_31_=0.0699	CL=BSA×(34.5-0.254×AGE)/35.6	VPC	NONMEM	Proportional/23.8
Patil *et al.* [25]	Three	ALT	CL_1_=18, CL_2_=6.49, CL_3_=29.7, V_1_=5.8, V_2_=2.65, V_3_ =468	No*	VPC and goodness of fit	NONMEM	Proportional/30.9
Crombag *et al.* [24]	Three	AGE, ALB, HEP	CL_1_=44, CL_2_=5.5, CL_3_=14, V_1_=12, V_2_=9.9, V_3_ =261	CL=BSA× (44-0.755×AGE+0.225×ALB) (1-0.334×HEP)	VPC and goodness of fit	NONMEM	Proportional/39.0
Xi *et al.* [45]	Three	ALB	CL_1_=57.2, CL_2_=14.1, CL_3_=8.63, V_1_=6.16, V_2_=11.8, V_3_ =660	CL=57.2× (ALB/42.8)^1.17^	Bootstrap and VPC	NONMEM	Proportional/27.0
Wang *et al.* [43]	Three	SCr, TBIL	CL_1_=37.82, CL_2_=12.63, CL_3_=30.30, V_1_=7.38, V_2_=5.61, V_3_ =911.97	CL=37.82× (Scr/63)^0.44^× (TBIL/10.3)^-0.63^	Bootstrap and VPC	NONMEN	Proportional/35.0

**Note:** PK: pharmacokinetic; PPK: population pharmacokinetic; 1/Tmax: one of the erythromycin breath test parameters; VPC: visual predictive check; NONMEM: nonlinear mixed-effects model; ALT: alanine aminotransferase; BMI: body mass index; BSA: body surface area; ALB: albumin; HEP: hepatic function (according to the original article); AAG: α1-acid glycoprotein; SCr: serum creatinine; TBIL: total bilirubin.*: The final model could not be established possibly due to lack of adequate number of patients with abnormal ALT.

**Table 3:** The clinical characteristics of enrolled patients.

**Characteristics** **No. of Patients (Samples)**	**Mean ± SD** **56 (112)**	**Median (Range)**
Age, year	54.95±10.31	55 (39, 73)
Height, cm	157.16±6.56	158 (139, 166)
Weight, kg	61.72±7.55	62 (50, 75)
BMI, kg/m^2^	25.04±3.13	24.67 (19.78, 31.2)
BSA, m^2^	1.73±0.10	1.73 (1.51,1.88)
TP, g/L	68.52±6.9	68.3 (36.7, 84.1)
ALB, g/L	40.59±3.32	40.4 (33.4, 49.1)
ALT	31.80±19.36	30 (9, 102)
AST	25.32±8.87	24 (11, 59)
ALP	80.43±29.40	72 (40, 209)
TBIL	10.51±3.53	9.91 (4.72, 25.21)
DBIL	2.00±1.08	1.62 (0.73, 5.09)
SCr	62.97±14.53	61 (34, 108)

**Note:** BMI: body mass index; BSA: body surface area; TP: total protein; ALB: albumin; ALT: alanine aminotransferase; AST: aspartate aminotransferase; ALP: alkaline phosphatase; TBIL: total bilirubin; DBIL: direct bilirubin; SCr: serum creatinine.

**Table 4:** Prediction-based diagnostic results.

**Author, Year**	**MPE/%**	**F_20_/%**	**F_50_/%**	**-2LL**	**AIC**
Launay-iliadis *et al.*, 1995 [20]	-8.05	32.14	63.39	17.27	43.27
Bruno *et al.*, 1996 [16]	-2.69	32.14	62.50	20.71	46.71
Slaviero *et al.*, 2004 [21]	-12.26	29.46	64.29	42.73	60.73
Minami *et al.*, 2009 [22]	-6.7	30.36	63.39	-4.83	21.17
Zhang *et al.*, 2012 [44]	-8.69	31.25	66.96	292.72	318.72
Onoue *et al.*, 2016 [23]	-7.54	29.46	62.50	104.78	122.78
Crombag *et al.,* 2019 [24]	-10.5	35.71	69.64	8.50	34.50
Patil *et al.,* 2021 [25]	-11.74	29.46	66.07	22.13	48.13
Wei *et al.,* 2022 [4]	-2.85	33.04	60.71	2.21	20.21
Xi *et al.*, 2022 [45]	-5.23	32.14	63.39	2.76	28.76
Wang *et al.*, 2022 [43]	-6.82	27.68	63.39	-22.91	3.09

**Note:** MPE: median prediction error; F_20_/%: the percentage of prediction error ≤ ±20%, F_50_/%: the percentage of prediction error ≤ ± 50%; -2LL: -2xlog (likelihood); AIC: Akaike information criterion.

## Data Availability

The datasets generated and analyzed in this study are available from the corresponding author [Z.L.] upon reasonable request.

## LIST OF ABBREVIATIONS

ALB: Albumin
AAG: α1-Acid Glycoprotein
ALT: Alanine Aminotransferase
AST: Aspartate Aminotransferase
BMI: Body Mass Index
BSA: Body Surface Area
Cis: Confidence Intervals
DBIL: Direct Bilirubin
HEP: Hepatic Function Indices
PPK: Population PK
PK: Pharmacokinetic
SCr: Serum Creatinine
TBIL: Total Bilirubin
TP: Total Protein
VPCs: Visual Predictive Checks

## STANDARDS OF REPORTING

STROBE guidelines were followed.
